# Adherence to WHO vaccine storage codes and vaccine cold chain management practices at primary healthcare facilities in Dalocha District of Silt'e Zone, Ethiopia

**DOI:** 10.1186/s40794-022-00167-5

**Published:** 2022-04-20

**Authors:** Diriba Feyisa, Fikadu Ejeta, Temesgen Aferu, Oliyad Kebede

**Affiliations:** 1 Department of Medicine, College of Health Sciences, Salale University, Fitche, Ethiopia; 2 Department of Pharmaceutics and Social Pharmacy, School of pharmacy, College of medicine and Health Sciences, Mizan-Tepi University, Mizan-Aman, Ethiopia

**Keywords:** Vaccine cold chain handlers, Knowledge, Adherence, Practice, Vaccine cold chain management

## Abstract

**Background:**

The main elements of effective vaccine cold chain management at the immunization service delivery point are well-trained vaccine cold chain handlers, vaccine storage equipment, and appropriate vaccine management procedures. Vaccine cold chain handlers must have enough expertise to provide the correct vaccine at the right time, maintain vaccine potency, and minimize vaccination failures. The study assessed knowledge of vaccine cold chain handlers on vaccine cold chain management, adherence to the WHO vaccine storage codes and vaccine cold chain management practice at primary health facilities in Dalocha district of Silt'e zone.

**Method:**

Institutional-based cross-sectional study was done at twenty-eight primary health facilities. One hundred forty primary health workers were drawn from four health centers and twenty-four health posts operating in Dalocha woreda of Silt'e zone, SNNPR, Ethiopia. A self-administered questionnaires and on-spot observation checklists were adapted from the WHO and WHO-UNICEF-effective vaccine management assessment tools to collect data from cold chain unit of the primary healthcare facilities. Data were entered to EPI data version 3.1; exported and analyzed using SPSS version 22. Statistical analysis was carried out to determine the level of knowledge, adherence to WHO cold chain management guideline and vaccine handling practice. The relationship that the knowledge of primary healthcare workers, primary healthcare workers training status, primary healthcare facilities' adherence to WHO vaccine storage codes, and length of work experience of primary health care workers have with the vaccine management practice were also explored

**Result:**

Above Half (54%) of the respondents have satisfactory knowledge of vaccine cold chain management. One hundred (71.4%) vaccine cold chain handlers did point correctly to the recommended range of temperature (2°C -8°C) for vaccine storage. Around two-thirds (63.6%) of them were aware of the twice-daily temperature recordings. Nearly half, (46.2%) of primary healthcare facilities have experienced poor adherence to the WHO storage practice codes. Around three-fifths of the observed primary healthcare facilities have registered undesirable vaccine management practices. The primary healthcare workers who received training on vaccine cold chain management (χ2 = 0.058, *p*=0.015), served at primary health care facilities for more five years (χ2 =18.545, *p*≤0.001), shown good adherence to WHO vaccine storage code (χ2 =18.545, *p*≤0.001), have sufficient knowledge on vaccine cold chain management (χ2=4.210, *p*≤0.031) were all significantly associated with desirable vaccine cold chain management practice.

**Conclusion:**

There is a gap in vaccine cold chain handlers’ knowledge about vaccine cold chain management and less than desirable adherence to WHO vaccine storage codes at primary healthcare facilities in Dalocha district. The majority of the observed primary health facilities have registered poor vaccine management practices. Everyone who has a stake in the cold chain management of vaccines should do their share, individually and collectively, to guarantee that everyone reaps the benefits of an effective cold chain.

## Introduction

Vaccinations are widely regarded, as one of the most effective and safest methods of public health primary prevention. On the other hand, Vaccine cold chain management is essential for reaping the full benefits of immunization in terms of increasing vaccination coverage and, more importantly, ensuring that tracer vaccines reach the intended recipient [1]. The World Health Organization (WHO) has stated that as much as 25% of all vaccine products reach their destination in a degraded state. Problems related to cold chain occur in every country that has had surveillance for vaccine temperature monitoring; meaning it may certainly not only happen in developing countries [2]. The Center for disease control (CDC) has estimated that each year, 300 million pounds worth of vaccines alone are destroyed globally due to improper storage and distribution [3]. In 2011, 2.8 million doses of vaccines were lost in 5 countries due to cold chain failures [4]. Maintaining the cold chain is an essential part of a successful immunization program as the immunological potency of vaccines could be compromised upon exposure to extreme temperatures [1, 5, 6]. Vaccine cold chain maintenance is the cumulative action achieved from materials, equipment, and techniques required to maintain vaccine temperatures between +2oC to +8oC during transportation, distribution, and storage until it reaches the recipient [1, 7, 8].

Vaccines are biological products that contain antigens capable of inducing a specific and active acquired immune response in the body and have been used for centuries to immunize individuals against pathogenic organisms to prevent the associated disease [9]. Vaccines are more exposed to the freezing temperature than heat damage at every level of the cold chain. Any loss of potency in a vaccine is permanent and irreversible [10].

Although the cold chain is commonly thought to prevent vaccine inactivation due to heat exposure, it is also critical to highlight potency loss due to freezing temperatures [11, 12]. Vaccines such as OPV, measles, varicella, oral typhoid are unstable to heat, while other vaccines such as DTP, HepB, and TT are sensitive to freezing [13, 14]. Most of the vaccines lose their potency within a short time when exposed to room temperature; that cold chain is an essential component for maintaining the quality of vaccine. So care must be taken to see that the vaccines do not lose their potency, before the date of expiry, by maintaining cold chain [15, 16].

Poor understanding of the dangers of vaccine freezing contributes to the weakness of the existing cold chain in many countries. Emphasis has long been placed on keeping vaccines cold, with less attention devoted to the prevention of vaccine damage from freezing. Freezing of vaccines in the cold chain is commonplace, potentially resulting in the widespread delivery of vaccines whose potency has been compromised [17]. Poor vaccine cold chain management practice could result in cold chain handlers’ inability to recognize that the potency of the vaccine has been compromised. This may lead to the inadvertent administration of sub-potent vaccines, which increases the risk that beneficiaries are not fully protected from disease [18–22].

Vaccine cold chain management is the main challenge in developing countries, including Ethiopia. A previous study indicated that only about 51.2% of health centers fill their temperature charts systematically twice a day as recommended [1]. The quality of vaccination services, which is critical for disease reduction, is being neglected due to inadequate knowledge, as vaccine cold chain handlers appear to be more concerned with ensuring vaccine coverage than maintaining vaccines in the cold chain [23–25].

Vaccine cold chain handlers are the most crucial personnel at a cold chain point as their correct knowledge and cold chain practices, vaccine management, and handling are immensely vital for the success of the Universal Immunization Programme. Hence, it is very pertinent that they are trained and supervised regularly to ensure the efficient practice of cold chain management at immunization service delivery points. In addition to training and supportive supervision of Vaccine cold chain handlers, logistic materials and tools for monitoring storage temperature (thermometers, temperature logging charts among others) should be available at healthcare facilities [26].

Although more than five decades have passed since Ethiopia launched EPI, many Ethiopian still suffer from vaccine-preventable diseases. One out of five children is still unprotected against vaccine-preventable illness [1, 21, 25, 27]. The Ethiopian government is working hard to improve vaccination coverage and the quality of the immunization program aligns with GAVI's strategy and the SDGs. The GAVI's strategy for 2021–2025, the vaccine goal phase-5, aims to bring a much stronger focus on reaching those most marginalized by strengthening primary health care systems, building and sustaining community demand using innovation to ensure that immunization services reach these children. The SDGs with 'leave no one behind' and 'reach the farthest behind first' principles will do the same [28]. The effective vaccine cold chain management at primary health facilities has a prominent role in realizing both the universal immunization component of the Ethiopian health sector transformation plan and routine immunization improvement plan [29]. The often neglected issue requiring close assessment is the knowledge of the people charged with ensuring an uninterrupted cold chain at the cold chain unit. Inadequate knowledge of cold chain management could lead to improper handling of vaccines and would result in altered potency of vaccines [30]. Primary healthcare facilities in developing countries face this risk due to the constraints of unsustainable electricity supply, inadequate and improperly set up storage facilities, perceived poor supervision and monitoring of cold chain management by the regulatory authority and traceability [31–34]. Ethiopia is not the exception to this reality. Primary healthcare facilities in the Dalocha District, like other health facilities in Ethiopia, operate under unreliable power sources and inadequate material, financial, and human resources that hinder the effective provision of essential immunization services to its’ vulnerable segment of the population. In addition, there is no evidence indicating that similar studies were conducted previously in Dalocha districts of Silt'e zone in Southern nation national and people’s regional state of Ethiopia regarding the vaccine cold chain management practice. With this background, this study assessed the knowledge of vaccine cold chain handlers, adherence of primary healthcare facilities to WHO vaccine storage codes, and vaccine cold chain management practice at primary healthcare facilities at Dalocha district of Silt'e zone, Ethiopia.

## Methods

### Study area

The study was conducted in Dalocha district, Silt'e zone of SNNPs region, Central Ethiopia. Dalocha district is one of the ten districts located in the Silt'e Zone and bounded on the south by Sankurra, the west by Wulbareg, the north by Silte, and the east by Lanfro. Dalocha town is the districts’ capital, located about 182 Kilometer from the Ethiopian capital, Addis Ababa. The ajority (92.4%) of the population residing in the Dalocha ditrict are rural dwellers. The district is between the highland and rift valley of the Ethiopia with an altitude of 1500 to 2000 meters above sea level.

### Study design and population

A facility -based cross-sectional study was conducted from January 3, 2021 to February 5, 2021. One hundred and forty vaccine cold chain handlers working in four health centers and twenty four health post were included in the study.

### Sample size determination and sampling technique

The sample size required for the study was calculated using single population proportion formula for cross-sectional study. n= $\frac{{\boldsymbol{z}}^{\mathbf{2}}\times \boldsymbol{p}\left(\mathbf{1}-\boldsymbol{p}\right)}{{\boldsymbol{d}}^{\mathbf{2}}}$ **;** Where: n = sample size**;** P = 0.5 maximum population proportion, since no previous studies found around study area; d = margin of sampling error tolerated, 0.05**;** Z = the standard normal value at confidence interval of 95% = 1.96**;** Therefore, ni = [(1.96)^2^ 5(1-0.5)]\(0.05)2 =384.16≈ 384**.** The reduction formula was used to determine final sample size. nf=ni/(1+ni/N)=384/(1+384/205)=133; Where ni=384, *N*=total number of primary healthcare workers (205 in primary healthcare workers at primary health facilities) and 15% allowance=20. The final sample size was calculated to be 153 using finite population correction formula as target population finite (N=205), which is advisable, when the target population is less than10,000 [35–38]. The stratified sampling method was used to recruit samples. Vaccine cold chain handlers were stratified based on their primary health facilities, then selected proportionally from each primary health facility.

### Main study variable Measurement

Knowledge towards a cold chain management: a twenty item questionnaire covering general aspects, good vaccine care (freeze sensitive vaccines, temperature-sensitive vaccines, vaccine vial monitor (VVM), shake test, time of use of a reconstituted vaccine, vaccine requiring diluents, and multi-dose vaccine vial policy (MDV-VP) and FEFO principle of vaccine management), good refrigerator care (conditioning of ice packs and refrigerator maintenance), temperature readings and monitoring (cabinet temperature range of ILRs and DFs) were used to assess vaccine cold chain handlers’ knowledge.

Adherence to WHO vaccine storage codes: When BCG and measles vaccines are placed in the lower basket (code-1), T-series and Hepatitis B vaccines are placed in the upper right basket (code-2), and diluents, returned partially used and unused vials are placed in the upper left basket (code-3), the compliance with WHO Vaccine Storage Practices is considered good. It is fair if at least two of three codes are practiced and graded as poor storage practice if only one code is practiced [4].

Vaccine cold chain management practice: vaccine cold chain management practice was also another main study variable, assessed on a fifteen point scale containing questions on vaccine handling practice.

### Data collection instruments

Data were collected using a self-administered questionnaire and on-spot observation checklists adapted from WHO and WHO-UNICEF-effective vaccine management assessment tool (WHO-UNICEF-EVMAT) to capture relevant information regarding practice and adherence of health facilities to the effective cold chain management [39, 40]. On-spot observation checklist was used to evaluate the availability of cold chain equipment adherence to WHO vaccine storage codes and vaccine management practice at primary health facilities. Data abstraction tools were prepared in English then pretested in health facilities out of the study area (at two health centers at Siltei woreda). The pharmacists were recruited, trained, and collected the data under the supervision of the principal investigator. Data were collected from the study participants; Nurses (clinical, pediatric, and neonatal), Health officers, Midwifery, pharmacy personnel (Dispenser and pharmacy storekeeper), and Health extension workers working at primary healthcare facilities and willing to participate in the study at the time of data collection.

### Data processing and analysis

Data were entered and cleaned by using Epi data version 3.1; thereafter, exported to SPSS Version 22 for further analysis. Data analysis was conducted to describe the nature of the data. The level of significance was set at *p* < 0.05. The results were presented in tables and figures.

### Data Quality Assurance

Training on data collection procedures were given to the data collectors for two days. Data collection tools were pretested. TA and OK reviewed tools for completeness, FE and DF supervised the data collection process.

### Ethical Considerations

The official letter of study permission was obtained from the school of pharmacy, College of Health Sciences, Mizan-Tepi University. A Letter of the permission obtained from Silt'e zone health department was presented to Dalocha Districts and primary healthcare facilities under its umbrella. Written informed consent was obtained from study participants before the data collection after the objectives of the study were made clear to them.

### Operational definition of terms

#### Cold chain handler

The key person for maintaining, monitoring and managing of cold chain and responsible for safe storage of vaccine in their respective units of primary healthcare facilities. They play an crucial role in maintaining an undisrupted cold chain as they are the last point of contact between the vaccines and the recipient [1].

#### Properly maintained refrigerator

The refrigerator with adequate air circulation between the vaccine boxes, vaccine kept only on the refrigerator shelves and not in the door or bottom drawer, and no food or drink stored in the refrigerator.

#### Vaccine cold chain handlers’ knowledge

The mean value of the correct responses (“YES”) that individuals received out of the 20 items used to determine their level of knowledge Vaccine cold chain handler who scored 50% and below were labeled as having unsatisfactory knowledge, while those who scored above 50% were labeled as having a satisfactory knowledge .

#### Acceptable/good/ vaccine cold chain management practice

Primary health facilities with scores of 10 and above (75%) were then categorized as having good practice or acceptable.

#### Desirable/optimal/vaccine storage

Vaccine storage is said to be desirable/optimal when primary healthcare storage facilities met more than 70% storage condition criterion/parameters of cold chain storage [41].

## Results

### General characteristics of respondents

A hundred-forty, (91.5%) of the vaccine cold chain handlers responded to the self-administered questioners. Seventy-six (54.6%) were female. Ninety-three (66.4%) were diploma holders, and 51(36.4%) were nurses by profession. Fifty-one (36.4%) have a service year ranging from 6 to 10 years with a mean and SD of 5±4 years of service (Table 1).

**Table 1:** General characteristics of Respondents at Primary Healthcare Facilities of Dalocha District of Silt'e Zone, Ethiopia; January 2021

Variable (*N*=140)		Frequency	***Percent***
Gender	Male	64	45.7%
	Female	76	54.3%
Level of education	Bachelor degree	47	33.6%
	Diploma	93	66.4%
Position	Pharmacy	6	4.3%
	Midwifery	19	13.6%
	Public health	26	18.6%
	Nurse	51	36.4%
	Other*	38	27.1%
Years of service	<5 years	89	66.6%
	5 years and above	51	36.4%

Other* = Store keeper and Health extension worker

### Training and supervision

Slightly above half of the respondents (51.4%) did not receive training. Among those who received training, above half (57.4%) of them have received the pre-service type of training. Only one-third (31%) have trained as recently as six months among those who received the in-service training. None of the respondents reported receiving supportive supervision from the district health management (Table 2).

**Table 2:** Training and supervision of the vaccine cold chain handlers’ at primary healthcare facilities of Dalocha District of Silt'e Zone, Ethiopia; January 2021

Training and supervision of respondents		Frequency	Percentage
Training	Yes	68	***48.6%***
	No	72	*51.4%*
Types of training	Pre-service	39	57.4%
	In-service	29	42.6%
Duration of in-service training	Once	11	7.8%
	Twice	12	8.6%
	Thrice	6	4.3%
Time of last in-service training	< 6 month	9	31%
	>6 month	20	68.9%
Supportive supervision of district health office in one month prior data collection	Yes	0	0
	No	140	100%
Feedback of supportive supervision	Yes	0	0
	No	140	100%

### Availability of infrastructure, cold chain equipment and resources

All primary health centers had a fuel generator, while half of them had fuel refrigerators and at least one functional refrigerator. Twenty-two of 24 health posts have a solar panel and have at least one working refrigerator. The majority of the public health facilities did not have cold chain follow-up employees assigned to them. One of the four health centers has a car/motorbike for transporting vaccinations in the event of a refrigerator power failure (Table 3).

**Table 3:** Infrastructure and cold chain equipment/resource availability at primary healthcare facilities of Dalocha District of Silt'e Zone, Ethiopia; January 2021

Characteristic (*N*=28)		Frequency (%)			
		Health center (*N*=4)		Health post (*N*=24)	
		Available	Not available	Available	Not available
Functional refrigerator		4 (100%)	0	22 (91.6%)	2 (8.4%)
Presence of power source	Electricity	4 (100%)	0	22 (91.6%)	2 (8.4%)
	Generator	2 (50%)	2 (50%)	0	24 (100%)
	Solar panel	1 (25%)	3 (75%)	22 (91.6%)	2 (8.4%)
	Kerosene	1 (25%)	3 (75%)	4 (16.7%)	20 (83.3%)
Functional car/motorbike in the facilities to use in case of refrigerator failure		1 (25%)	3 (75%)	0	24 (100%)
Spare parts for minor cold chain maintenance		0	4 (100%)	0	24 (100%)
Cold box		2 (50%)	2 (50%)	4 (16.7%)	20 (83.3%)
EPI guidelines or manual		1 (25%)	3 (75%)	0	24 (100%)
Temperature recording sheet		2 (50%)	2 (50%)	10 (41.7%)	14 (58.3%)
Vaccine order form		3 (75%)	1 (25%)	0	24 (100%)
Ice pack		2 (50%)	2 (50%)	18 (75%)	6 (25%)
Vaccine carrier		1 (75%)	3 (25%)	19 (79.2%)	5 (20.8%)

Twenty-six out of 28 primary healthcare facilities have at least one functional refrigerator at their respective cold chain units. Most (80.7%) of those primary healthcare facilities used only domestic type of refrigerators while less than one-tenth (7.7%) of primary health care facilities visited used ice-lined refrigerators.

### Knowledge of vaccine cold chain handlers regarding Vaccine cold chain management

The majority (71.4%) of the respondents correctly mentioned the recommended range of temperature (2°C -8°C) for vaccine storage, while 89(63.6%) of the respondents recorded the temperature twice daily. Sixty-nine (49.3%) of the respondents kept records of received, stored vaccines, and were aware of the vaccine inventory management using the EEFO principle. Seventy-eight (55.7%) respondents updated the stock register once within one working day. More than half of respondents correctly name the most freeze-sensitive vaccine, while less than half name the most heat and light-sensitive vaccines. Slightly above half of the respondents were aware of the importance, process, and interpretation of Vaccine Vial Monitor. Seventy-nine (56.4%) of respondents were aware of the reason behind doing the “Shake Test”. Two-third of the respondents know about good refrigerator care while only one-third are aware of cold chain Preventive maintenance and cold chain contingency plan. There was a significant variation in knowledge between vaccine cold chain handlers who got training and those who did not train regarding temperature-sensitive to vaccines, the recommended temperature range correct order of storing vaccines, numbers of times to updates the stock register, importance’s regular defrosting refrigerators and about open vial policy. Overall, 75 (54%) of primary health care workers had a satisfactory knowledge regarding cold chain management (Table 4).

**Table 4:** Knowledge of vaccine cold chain handlers on vaccine cold chain management at primary Healthcare facilities of Dalocha District of Silt'e Zone, Ethiopia; January 2021

No.	Knowledge of vaccine cold chain handlers on vaccine cold chain management operation (*N*=140)	Frequency (percentage)			*P*-value*
		Correctly responded	Training status		
			Trained	Not Trained	
1	Aware about the importance and interpretation VVM	71 (50.7%)	28 (20%)	43 (30.7%)	0.6487
2	Correctly name DTP-Hib, Hb, TT as the list of vaccines that should be protected from sub-zero temperatures	83 (59.3%)	54 (38.6%)	29 (20.7%)	0.0025
3	Correctly name OPV and Measles as the most heat sensitive vaccines	59 (42.1%)	40 (28.5%)	19 (13.6%)	0.0011
4	Correctly name BCG and Measles as the most light sensitive vaccines	62 (44.3%)	43 (30.7%)	19 (13.6%)	0.7619
5	Identified the recommended temperature range for storing vaccines between +2^o^C to + 8 ^o^ C	100 (71.4%)	61 (43.6%)	39 (27.8%)	0.0025
6	Knows and correctly explained about Open Vial Policy; The discarding period of reconstructed vaccine vial.	64 (45.7%)	42 (30%)	22 (15.7%)	0.0031
7	Correctly identified the diluents of BCG and Measles Vaccine	67 (47.8%)	41 (29.3%)	26 (18.5%)	0.601
8	Aware about the “Shake Test” and it’s interpretation	79 (56.4%)	55 (39.3%)	24 (17.1%)	0.0325
9	Knows about the conditioning of ice-packs	82 (58.6%)	44 (31.5%)	38 (27.1%)	0.0564
10	Knows about the twice daily recording of the temperature log book	89 (63.6%)	54 (38.5%)	35 (25.1%)	0.0203
11	Tells the numbers of times to updates the stock register	78 (55.7%)	51 (36.5%)	27 (19.2%)	0.0014
12	Aware about importance’s regular defrosting refrigerators	109 (77.9%)	73 (52.2%)	36 (25.7%)	0.0012
13	Knows the correct order of storing vaccines in ILR	87 (62.2%)	60 (42.9%)	27 (19.3%)	0.0039
14	Knows about the contingency plan and what to do just incase	63 (45%)	33 (23.7%)	30 (21.3%)	0.3562
15	Know the Correct placing thermometer inside deep freezer & ILR; in the middle level	76 (54.3%)	43 (30.7%)	33 (23.6%)	0.1630
16	Correctly indicated the length of time to keep vaccines in vaccine carrier between 3-18 hr.	68 (48.6%)	32 (22.9%)	36 (25.7%)	0.9211
17	Knows about the Good refrigerator care; avoid placing nearby sunlight, stove or microwave	94 (67.1%)	38 (27.1%)	56 (40%)	0.6179
18	Aware about cold chain equipment maintenance; Preventive maintenance every month fixed day	53 (37.9%)	25 (17.9%)	28 (20%)	0.8314
19	Aware about need of temperature monitoring on holiday	87 (62.1%)	44 (31.4%)	43 (30.7%)	0.4177
20	Aware about the vaccine inventory management and EEFO principle	69 (49.3%)	36 (25.7%)	33 (23.6%)	0.0532
**Level of primary health care workers’ knowledge regarding vaccine cold chain management at primary health facilities of Dalocha district**				**Frequency (percentage)**	
Level of primary health care workers knowledge regarding vaccine cold chain management	Satisfactory			75 (54%)	
	Unsatisfactory			65 (46%)	

* *P* value obtained from Fisher Exact Test.

### Primary healthcare facilities’ Adherence to WHO vaccine storage codes

At the time of visit, out of 26 primary health facilities with functional refrigerators, 12 (46.2%) of them experienced poor storage practices followed by 10 (38.5%) with fair storage practice and 6 (23.1%) with good storage practice as per WHO cold chain storage codes (Table 5).

**Table 5:** Primary Healthcare facilities’ Adherence to WHO vaccine storage codes in Dalocha District of Silt'e Zone, Ethiopia; January 2021

Item No.	Primary health workers Adherence to WHO vaccine storage code (vaccine storage practice in the refrigerators)	Frequency (percentage)	
		Yes (%)	No (%)
1	Adhere to Code 1:-BCG and measles vaccine in lower basket (*n*=26)	10 (38.5%)	16 (61.5%)
2	Adhere to Code 2: T-series and Hepatitis B vaccine in upper right basket (*n*=26)	12 (46.2%)	14 (53.8%)
3	Adhere to Code 3: diluents, returned partially used and unused vials in upper left basket (*n*=26)	7 (26.9%)	19 (73.1%)
**Level of adherence to WHO vaccine storage code**		**Frequency (percentage)**	
4	Good adherence to WHO vaccine storage code (*n*=26)	6 (23.1%)	
	Fair adherence to WHO vaccine storage code (*n*=26)	10 (38.5%)	
	Poor adherence to WHO vaccine storage code (*n*=26)	12 (46.2%)	

### Vaccine Cold chain management practice

All health centers have kept the temperature of the refrigerator within the recommended range (+ 2 °C to + 8 °C), three of four (75%) have stored cold chain products separate from other items; while vaccines arranged based on first to expire first-out (FEFO) two of them (50%) had one functional thermometer. All primary health centers had correct placement of ice-packs inside the freezer while only one (25%) health center has had a standby power supply. Half of (two) health centers had cold chain product packing areas protected from direct sunlight, and one of the health centers had vaccine LMIS reporting and requisition. The majority of observed health centers did not record temperature twice daily; the VVM status of each vaccine was not recorded and expired vaccines were not presented in the refrigerator (Table 6).

**Table 6:** Vaccine cold chain management practice at primary Healthcare facilities of Dalocha District of Silt'e Zone, Ethiopia; January 2021

Item No.	Adherence to WHO vaccine cold chain management practice guideline (health workers practice)	Frequency (percentage)	
		Yes (%)	No (%)
1	The refrigerator is dedicated for storage of cold chain pharmaceuticals (*n*=26)	10 (39.5%)	16 (61.5%)
2	The refrigerator have sufficient storage capacity (*n*=26)	23 (88.5%)	3 (11.5%)
3	Vaccines are properly arranged in refrigerator (*n*=26)	9 (34.6%)	17 (65.4%)
4	Vaccine management following EEFO principle (*n*=28)	12 (42.9%)	16 (57.1%)
5	The expired vaccines are not present in refrigerator (*n*=26)	19 (73.1%)	7 (26.9%)
6	The frozen vaccines are not present in refrigerator (*n*=26)	20 (76.9%)	6 (22.1%)
7	Refrigerator temperature maintained between 2-8^0^C (*n*=26)	23 (88.5%)	3 (11.5%)
8	Functional thermometer present in the refrigerator (*n*=26)	8 (30.8%)	18 (69.2%)
9	The refrigerator T^0^ recorded twice daily as reflected by temperature registration sheet on the door of refrigerator (*n*=26)	9 (34.6%)	17 (65.4%)
10	The refrigerator is protected from direct sun light to aid good refrigerator care (*n*=26)	12 (46.2%)	16 (57.1%)
11	All vaccine receipt and transactions is recorded (*n*=28)	9 (32.1%)	19 (67.9%)
12	The VVM status of vaccine recorded for each vaccine in vaccine status registration book (*n*=28)	3 (10.7%)	25 (89.2%)
13	Vaccine LMIS (Logistic management information system) reporting and requisition form done in the last month (*n*=28)	7 (25%)	21 (75%)
14	Presence of standby power supply beside the main sources (*n*=28)	2 (7.1%)	26 (92.9%)
15	Cold chain pharmaceutical are stored on doors of the refrigerator shelf (*n*=26)	15 (57.7%)	11 (42.3%)

### Level of vaccine cold chain management practice

**Fig. 1:**
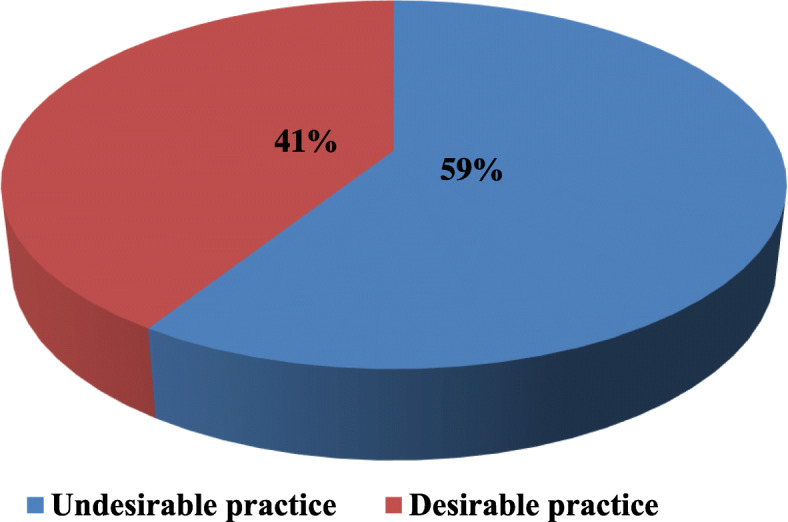
Vaccine cold chain management practice observed at primary Healthcare facilities in Dalocha District of Silt'e Zone, Ethiopia; January 2021

Two-fifth (41%) of observed primary health facilities has experienced undesirable vaccine cold chain management practice (Fig. 1).

### Relationships between Independent Variables and Vaccine cold chain management practice

The relations between vaccine cold chain handlers vaccine cold chain management training, years of service, adherence to the WHO vaccine storage code, knowledge of primary healthcare workers, and vaccine cold chain management practice were investigated. Training on vaccine cold chain management, years of service and knowledge of health care professional were associated with vaccine cold chain management practice. The primary healthcare workers who received training on vaccine cold chain management (χ2 = 0.058, *p*=0.015), served at primary health care facilities for more five years (χ2 =18.545, *p*≤0.001), shown good adherence to WHO vaccine storage code (χ2 =18.545, *p*≤0.001), have sufficient knowledge on vaccine cold chain management (χ2=4.210, *p*≤0.031) were all significantly associated with desirable vaccine cold chain management practice (Table 7).

**Table 7:** Relationships between Independent variables and vaccine cold chain management practice at primary Healthcare facilities in Dalocha District of Silt'e Zone, Ethiopia; January 2021

Independent Variables	X^**2**^	***P***-Value
Primary healthcare workers Training on vaccine cold chain management	3.058	0.015
Primary healthcare workers years of service	10.53	0.038
Adherence to WHO vaccine storage code	18.89	0.021
Knowledge of Primary healthcare workers on vaccine cold chain management	6.125	0.001

## Discussion

Vaccination has to be sustained as a high priority to reduce the incidence of all VPDs. Although Government of Ethiopian is working hard in improving vaccination coverage; the country is facing a phase-out of donor support for several programs aimed at improving vaccination coverage and quality improvement in underserved administrative regions and zones. The Routine Immunization Improvement Plan that currently supported by GAVI could be critical in improving vaccination coverage among children who are currently not reached by immunization services. The effective vaccine cold chain management at primary health facilities has a prominent role toward the realization of universal immunization component of the Ethiopian health sector transformation plan as well as routine immunization improvement plan.

In the comprehensive multi-year plan 2016–2020 of the Ethiopia National Expanded Programme on Immunization, the Ministry of Health identifies inequities in access to immunization services, as well as regional disparities, as major weaknesses in immunization service delivery [29]. Vaccine cold chain management, ensuring the optimal potency of vaccines to maximize the resulting efficacy of vaccination, at primary health facilities is one of the most contributing factor to this major weaknesses in immunization service delivery [1, 31]. Hence, proper maintenance of the cold chain is vital in maintaining the effectiveness of vaccines in routine immunization. It is also crucial to assess the level of primary healthcare facilities' adherence to the WHO vaccine storage codes, and the status of vaccine cold chain management practices at primary healthcare facilities and knowledge of vaccine cold chain handlers’ in Dalocha district of Siltei Zone, Ethiopia. The vaccines’ cold chain is assumed to be at the highest risk, particularly at this area as Dalocha district of Siltei Zone is one the rural districts in Ethiopian region with unreliable power supply, transportation, and skilled human-power and with underdeveloped Cold chain maintenance. Ensuring the optimal potency of vaccines at primary healthcare facilities to maximize the resulting efficacy of vaccination, is one of the most contributing factors to the main weaknesses in immunization service delivery [31].

The study revealed that, among those who received the in-service type of training, only one-third (31%) of vaccine cold chain handlers trained as recently as six months. This is lower than the figure from the study done in Cameron [42], Tanzania [43], Malaysia [44], and India [45]. Most donor agency focused on pre-service training and In-service training is currently overlooked. This might attributable to the Covid-19 pandemic and its health and economic hangover and impact. Cold chain and vaccine management are essential components for improving the quality of immunization services [1]. Careful attention to vaccine storage and handling is crucial to ensure the optimal potency of vaccines to maximize the resulting efficacy of vaccination [31]. Proper maintenance of the cold chain is vital in maintaining the effectiveness of vaccines in routine immunization. It is critical to know the prominence of primary healthcare workers' knowledge regarding vaccine cold chain management, the level of primary healthcare facilities' adherence to the WHO vaccine storage code, and the status of vaccine cold chain management practices in primary healthcare facilities in Dalocha district of Silt’e Zone, Ethiopia. This study showed that 54% of health professionals had satisfactory knowledge regarding vaccine cold chain management. This figure is nearly consistent with a study done in the Bale Zone [21], Ezha district of Gurage zone [46], central Ethiopia [47], and Tanzania [43]. However, It is higher than the figure observed from the study done in the East Gojam zone of Ethiopia, where 38.3% of the respondents had sufficient knowledge about vaccine cold chain management [27]. It is much lesser than a figure from the studies conducted in Nigeria [26], and Malaysia [48] that reported 83.9% and 78.2% of respondents have good knowledge of vaccine cold chain management. This discrepancy might be due to a difference in primary healthcare workers' motivation, qualification, and district health management support. In this study, about 71.4% of the respondents knew the recommended temperature range for vaccine storage. This finding is consistent with a study done in the Ezha district of the Gurage zone [46], East Gojam zone of Ethiopia [27], and Western India [8]. But, this result is higher than a study conducted in Cameroon [32] and Nigeria [26]. Thirty-nine percent of the primary health workers knew the proper compartment of the measles vaccine. This finding is nearly similar to a study done in the Jimma zone in which 62.3% of health professionals knew the compartment of the measles vaccine [1]. This is lower than the studies done in Central Ethiopia [49], the Ezha district of the Gurage zone [46], and Mozambique [19]. This discrepancy might be due to an absence or insufficient training, workload, or staff turnover. More than one-third (42.4%) of primary health workers were able to list vaccines that are most sensitive to heat. This figure is consistent with a study done in central Ethiopia [47] and the Ezha district of the Gurage zone of Ethiopia [46]. But, It is much lesser than a study conducted in Malaysia [48]. Around three-fifth (59.3%) of primary health workers knew the vaccines that are most sensitive to cold, and this finding is higher than studies done in central Ethiopia [47] and North-Central Nigeria [50]. But, the figure is lesser than a study conducted in Malaysia [48]. This difference might be attributed to disparity in the level of education and provision of in-service training among primary health workers in the countries.

In the present study, all primary health centers and most of the health posts (91.6%) had a functional refrigerator. This finding is nearly similar to the figure from a study conducted in the North-West Region of Cameroon in which 95.1% of health facilities had a functional refrigerator with working thermometer [23]. The disparity may be attributable to socioeconomic differences across the nations and the research’s objectives; as a study conducted in North-Western Cameroon was a pilot study. However, it is higher than a study done in central Ethiopia in 2016 in which only 19% of the facilities had a functional refrigerator [49]. This difference reflects post millennium country’s political commitment to realize universal immunization program via availing functional cold chain equipments that maintains vaccines in desirable condition before reaching at the arms vaccine recipients.

Proper storage and management of vaccines in a cold chain system are essential for immunization. Improper storage and poor management of cold chain equipment adversely affect the efficacy of vaccine thus immunization program. Thirty-nine percent of primary healthcare facilities kept vaccines with another cold chain pharmaceutical in their refrigerators. This finding was higher than the study conducted in the Ezha district of Gurage zone [46], central Ethiopia [49], Chandigarh [33], Qatar [51], and South India [20]. The variation might be due to disparity in the level of motivation, supervision, negligence of the primary healthcare workers, and poor knowledge vaccine cold chain handlers on the effect of repeatedly opening the fridge on vaccine potency.

This study identified the gaps related to vaccine cold chain management practice. There were inconsistencies in vaccine cold chain management practices, such as the availability of a backup power supply, the recording of damaged vaccines, the monitoring of temperature twice daily, and the recording of VVM status for each vaccine. There was also a gap in using the vaccine form for reporting and ordering vaccines. One-fourth of health facilities had a functional thermometer in their refrigerator. This finding is consistent with a study done in central Ethiopia [21]. however, it is lesser in comparison to Cameroon’s research results [42]. The finding of this study also revealed that only two-fifth (41%) of observed primary health facilities experienced undesirable vaccine cold chain management practices. This finding is lower than the study done in the Ezha district of the Gurage zone of Ethiopia [46], in 2 districts of Eastern India [34], southern Nigeria [24], and Nigeria [26]. This disparity might be due to the differences in primary worker training between the nations and cold chain equipment utilization that maintains the vaccine cold chain system. The success of cold chain management depends very much on the Health facilities' adherence to the WHO vaccine storage code. Twelve (46.2%) of primary health facilities followed poor WHO storage practice codes. This finding corroborates with the study done in public health facilities in the Jimma zone of Ethiopia [1], West Shewa zone of Ethiopia [52], Ghana [53], and India [7].

Training on vaccine cold chain management, years of service, adherence to the WHO vaccine storage code, and knowledge of primary health facilities were associated with vaccine cold chain management practice. Training on vaccine cold chain management, years of service and knowledge of health care professional were associated with vaccine cold chain management practice. The primary healthcare workers who received training on vaccine cold chain management (χ2 = 0.058, *p*=0.015), served at primary health care facilities for more five years (χ2 =18.545, *p*≤0.001), shown good adherence to WHO vaccine storage code (χ2 =18.545, *p*≤0.001), have sufficient knowledge on vaccine cold chain management (χ2=4.210, *p*≤0.031) were all significantly associated with desirable vaccine cold chain management practice. The figure corroborates with the study done in Nigeria [26], Cameron [32], and Ethiopia [54],

### Study Limitations

The study was conducted in primary healthcare facilities in one district. Hence, caution is needed in generalizing these results as the findings from this study only represent the primary health care facilities in the Dalocha district of the Silt'e zone. As a result, future research should replicate these findings with the large sample size and different studies to replicate data that may be used to generate information for health system operations management and extend the study’s findings to other healthcare settings.

## Conclusion and Recommendation

The information from this study will have more impact on the district healthcare administrator in this region. The study showed that the cold chain infrastructure, cold chain equipment, a record of power failure/cut, vaccine management practice, and adherence to WHO vaccine handling and storage codes at primary health facilities were less than desirable. The study indicated gaps among vaccine cold chain handlers in managing cold chain, as slightly above half have satisfactory knowledge. Only one-third of Primary healthcare facilities have good adherence to the WHO vaccine storage code. Three-fifth of observed primary health facilities has undesirable vaccine cold chain management practices. Undesirable vaccine Cold chain management practice in primary health care at the study area could compromise the potency of the vaccines and the general quality of the immunization services. Vaccines have been stored together with other items. The exposure of vaccines to abnormal storage conditions, the inability to implement the FEFO principle of vaccine inventory management, frequent opening of the refrigerator, and discarding based on VVM status change once it reaches the discard point were all undesirable practices observed in primary healthcare facilities. Therefore, the administration of vaccines that are exposed to abnormal storage conditions leads to immunization failure. Training on vaccine cold chain management, years of service, and knowledge of health care professionals were associated with vaccine cold chain management practice. The stakeholders involved in aiding the success of vaccine cold chain management should ensure that they play their parts individually and collectively to ensure that the dividends of an efficient cold chain are enjoyed by all and varied. There is a need for periodic assessment at all primary health facilities by healthcare authorities to ensure that the standardized system for maintenance of vaccine cold chain system is practiced by all health care professionals at the primary healthcare facilities. The regional and district healthcare manager (Dalocha district health office, Silte Zonal Health Department, S/N/N/Ps Regional Health Bureau and EPSA-Hawassa hub) needs to provide a means of updating the knowledge of primary health care workers about vaccine management through the provision of continuous in-service training, frequent monitoring, supervision, and giving feedback to their supervision for HEWs whose education is less than or equal to grade 12 at health post level, and other at the primary health center level. Vaccine cold chain handlers’ needs to maintain acceptable vaccine storage conditions by appropriately implementing the EPI-Standard operating procedure and placing contingency plans in place during a power outage or equipment failure. The sponsors and researcher should work toward developing a data base (software) available for region, ZHD, and EPSA which helps to track and control the cold chain equipments’ status and to monitor vaccine cold chain status during transportation and storage that enables the cold chain system to be more responsive and efficient. Future studies ought to be done at the region to investigate the differences in knowledge among vaccine cold chain handlers more concretely.

## Data Availability

The data sets generated and/or analyzed during the present study are available from the corresponding author on reasonable request.

## Abbreviations

BCG: Bacillus Calmette–Guérin
CCP: Cold Chain Product
CDC: Center For Disease Control
DPT: Diphtheria Pertussis Tetanus
DTP-Hib: Diphtheria Pertussis Tetanus-Hepatitis B
DFs: Deep Freezers
EPI: Expanded Program Immunization
EVMT: Effective Vaccine Management Tool
EPSA: Ethiopian pharmaceutical supply agency
FEFO: First Expiry First Out
FDRE: Federal Democratic Republic of Ethiopia
GAVI: The Global Alliance for Vaccines and Immunizations
HepB: Hepatitis B
HEWs: Health Extension Workers
ILRs: Ice-lined Refrigerators
LMIS: Logistic Management Information System
MDV-VP: Multi-dose vaccine vial policy
NGO: Non-Governmental Organization
OPV: oral polio vaccine
VPD: Vaccine Preventable Diseases
VVM: Vaccine Vial monitor
SDGs: Sustainable Development Goals
S/N/N/Ps: Southern Nation, Nationalities and Peoples’ region
TT: Tetanus toxoid
UNICEF: United Nations Children's Fund
WHO: World Health Organization
ZHD: Zonal Health Department
